# Green chemometric-assisted UV-spectrophotometric methods for the determination of favipiravir, cefixime and moxifloxacin hydrochloride as an effective therapeutic combination for COVID-19; application in pharmaceutical form and spiked human plasma

**DOI:** 10.1186/s13065-024-01168-5

**Published:** 2024-04-05

**Authors:** Eman A. Madbouly, Abdalla A. El-Shanawani, Sobhy M. El-adl, Ahmed S. Abdelkhalek

**Affiliations:** Department of Medicinal Chemistry, Faculty of Pharmacy, Zagazig University, Zagazig, Egypt

**Keywords:** Covid-19, Spectrophotometry, Chemometrics, Partial least square, Genetic algorithm

## Abstract

As pharmaceutical analysis progresses towards environmental sustainability, there is a growing need to enhance the safety and health conditions for analysts. Consequently, the incorporation of chemometrics into environmentally friendly analytical methods represents a promising approach. Favipiravir, cefixime, and moxifloxacin hydrochloride have been currently used in COVID-19 treatment. In this study, we develop spectrophotometric methods depending on chemometric based models to measure the levels of favipiravir, cefixime, and moxifloxacin hydrochloride in pharmaceutical preparations and spiked human plasma. It is challenging to determine favipiravir, cefixime, and moxifloxacin simultaneously because of overlap in their UV absorption spectra. Two advanced chemometric models, partial least square (PLS) and genetic algorithm (GA), have been developed to provide better predictive abilities in spectrophotometric determination of the drugs under study. The described models were created using a five-level, three-factor experimental design. The outcomes of the models have been thoroughly assessed and interpreted, and a statistical comparison with recognized values has been taken into consideration. The analytical eco-scale and the green analytical procedure index (GAPI) evaluation methods were also utilized to determine how environmentally friendly the mentioned models were. The outcomes demonstrated how well the models described complied with the environmental requirements.

## Introduction

**Fig. 1:**
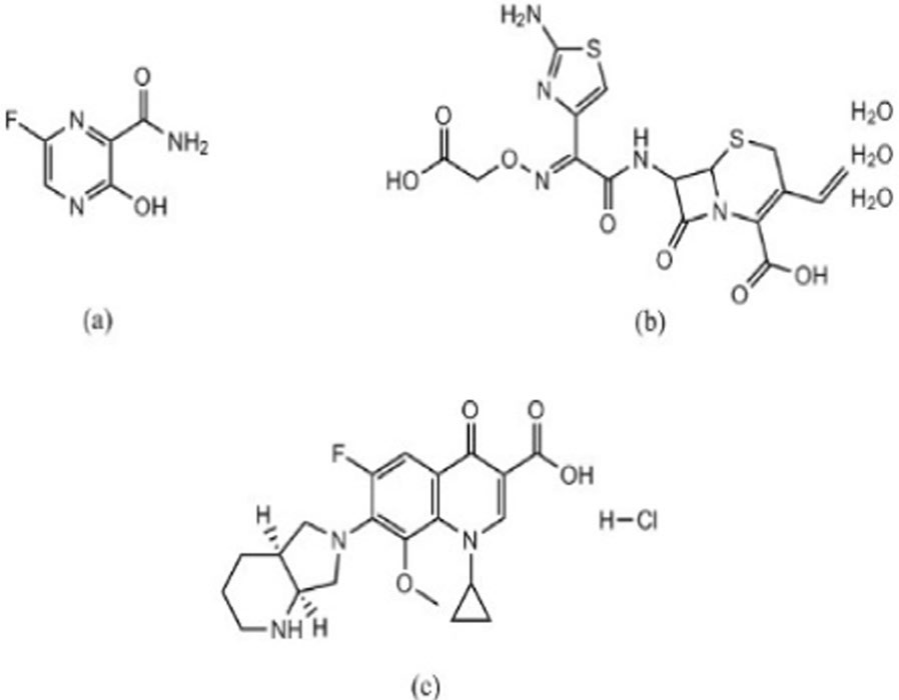
Structural formula of FPV (**a**), CEF (**b**) and MFX (**c**)

The coronavirus disease 2019 (COVID-19) outbreak has become a worldwide crisis due to the devastation it has caused and its rapid spread [1]. This disease is brought on by a novel infectious positive single-stranded RNA virus called SARS-CoV2, and it frequently comes with multiple cases of atypical pneumonia. Although there has been quick progress in developing SARS-CoV-2 vaccines, drug repurposing is still a crucial part of treating various illnesses [2]. Antivirals and antibiotics are mainly used in COVID-19 treatment. Favipiravir (FPV), Fig. 1a is a pyrazine carboxamide derivative. It is an analogue of purine nucleic acid that replaces guanine or adenine and hinders viral replication by preventing RNA-dependent RNA polymerase (RdRp). It is administered as a prodrug that, when phosphoribosylated intracellularly, can produce the active compound FPV ribofuranosyl-5B-triphosphate [3]. For the quantitative determination of FPV, various analytical approaches were reported, including liquid chromatographic [4–10], electrochemical [11–14], spectrophotometric [15–17], spectrofluorometric [6, 18] and densitometric [19, 20] methods.

Antibiotics are used to treat bacterial infections that coexist with COVID-19 infections or to exploit their possible antiviral properties. Cefixime trihydrate (CEF), Fig. 1b, is a semi-synthetic cephalosporin antibiotic of third-generation that is taken orally. It is an antibacterial agent that is used to treat bronchitis, and pneumonia. Cefixime’s antibacterial effect is due to its ability to prevent the formation of mucopeptides in the bacterial cell wall [21].

Moxifloxacin hydrochloride (MFX), Fig. 1c, is a fourth generation fluoroquinolone antibiotic. Its mechanism of action relies on inhibition DNA gyrase, also known as topoisomerase II, an enzyme that is necessary for the replication of bacterial DNA [22]. The combination of MFX and CEF has been approved by the FDA [23]. So, this combination can be used as adjuncts therapy in treating patients who have COVID-19. A survey of the literature indicates that the most popular analytical method for CEF/MFX analysis is high-performance liquid chromatography (HPLC) [24–26]. Nevertheless, the documented HPLC techniques have certain drawbacks, such as the unusual use of potentially harmful organic solvents in the mobile phase as acetonitrile as well as laborious separation processes. Additionally, choosing the right stationary and mobile phases is one of the crucial factors that needs to be precisely adjusted for the best peak resolution. On the other hand, spectrophotometric methods for drug analysis can eliminate the aforementioned issues with increased ease, effectiveness, and precision. Available spectrophotometric techniques used for CEF/MFX determination include mathematical manipulation techniques like first derivative, and first derivative of the ratio spectra [23, 27]. These techniques have drawbacks as well, such as inefficient data collection that could lower the throughput of analytical methodology. These approaches also have drawbacks since they are ineffective at gathering unnecessary data, which could lower the throughput of analytical methodologies due to wasteful data collection. Furthermore, when a data spectrum is analyzed using only one or two points, these methods are extremely sensitive to interfering factors because it is challenging to discern the analyte signal from an interferent. Moreover, every drug needs a calibration curve, and a number of tests are needed to choose the appropriate divisor for the next derivative of the ratio spectra [28, 29]. As a result, chemometrics has garnered a lot of interest lately as a successful post-processing method that can address the aforementioned drawbacks [30]. Partial least squares (PLS) and genetic algorithm partial least squares (GA-PLS) were popular two assisted chemometric spectrophotometric methods for the quantitative analysis of complex mixtures without any recommended need for a prior separation [31, 32].

In addition, no method for simultaneously evaluating FPV, CEF and MFX in co-formulation as co-administered drugs has been reported. This means that hospitalized inpatients require a method to determine those medications simultaneously in order to evaluate their therapeutic drug monitoring [33]. The aim of this study is to develop and validate two new multivariate chemometric methods (PLS and GA-PLS) for the simultaneous analysis of the cited drugs in bulk powder, pharmaceutical dosage forms and spiked human plasma. Also, this study aims to develop the first analytical method capable of estimating those co-administered drugs in the co-formulation while taking into consideration green analytical chemistry concepts. Several tools, including the analytical eco-scale [34] and the green analytical procedure index [35] were used to assess the models' level of greenness. Also, the models given showed superiority with the greenness characteristics in terms of the conventional green metric values. Through the integration of chemometric tools and their application with green assessment metrics, the authors aim to offer a promising challenge for accomplishing green goals.

## Experimental

### Chemicals

FPV (99.65%) pure powder was kindly supplied by Biophore India Pharmaceuticals Private Limited (Telangana, India). (CEF) (99.50%) pure powder was graciously donated by Kahira Pharmaceutical and Chemical Industrial Company-Cairo-Egypt. MFX (99.45%) pure powder was graciously donated by EVA Pharmaceutical Industrial Company (Cairo, Egypt). All of the chemicals were of analytical grade, the solvents were HPLC grade, and the water was freshly distilled throughout the entire process.

Favipiravir® Tablet (400 mg FPV/Tablet), manufactured by ZHEJIANG HISUN Pharmaceutical Company (batch number 23006020), was purchased from the Chinese market. Moxinow® Tablet (400 mg CEF & 400 mg MFX/Tablet) manufactured by Lupin Ltd (batch number 005G23OS), was purchased from the Indian market.

### Apparatus and software

A UV-1800 PC double-beam Shimadzu UV–Vis spectrophotometer, with UV probe software, was utilized. PLS and GA were implemented in MATLAB R2015a (8.5.0.197613) employing the PLS toolbox software version 2.1.

### Standard solutions

By dissolving 10 mg of each standard in 70 mL of distilled water in separate 100 mL volumetric flasks, and then bringing the volume to 100 mL with distilled water, three distinct stock solutions (100 μg/mL) of FPV, CEF and MFX have been obtained.

### Procedures

#### PLS and GAPLS models design

Arguably, one of the most important steps to improve the likelihood of obtaining representative and instructive data is to plan your experiments well. A partial five-level/three-factor factorial design would have been ideal for creating calibration and validation sets. In the beginning, twenty-five FPV, CEF and MFX mixtures were created and split into calibration and validation sets.

The calibration set was prepared using five concentration levels for each component to produce 13 laboratory-prepared mixtures with various concentrations ranges: 3–7 μg/mL for each of FPV, CEF and MFX.

The design’s central level is 5 μg/mL for each drug. To prevent any overfitting of the created models, a total of twelve combinations of the three medications under study were selected as the validation set. The calibration and validation sets’ concentrations were established using the partial factorial experimental design approach. The results are shown in Table 1.

**Table 1:** Concentrations of FPV, CEF and MFX mixtures used in the calibration and validation sets

No. of mix	Calibration set			No. of mix	Validation set		
	Concentrations, μg/mL				Concentrations, μg/mL		
	FPV	CEF	MFX		FPV	CEF	MFX
**1**	5	5	5	**1**	5	3	3
**2**	3	3	7	**2**	3	7	4
**3**	7	4	7	**3**	4	7	5
**4**	7	5	4	**4**	5	4	4
**5**	4	4	6	**5**	4	6	7
**6**	6	7	6	**6**	7	6	5
**7**	6	5	7	**7**	5	7	7
**8**	7	7	3	**8**	7	3	6
**9**	3	6	3	**9**	6	3	5
**10**	3	5	6	**10**	5	6	6
**11**	6	6	4	**11**	6	4	3
**12**	4	3	4	**12**	3	4	5
**13**	4	5	3				

### Application to pharmaceutical preparation

#### FPV

Ten Favipiravir® tablets (400 mg/tablet) were finely ground and weighted. A precise weight measurement was used to determine the appropriate amount of powder, equivalent to 10 mg of FPV. The powder was then transferred to a 100-mL volumetric flask, and the volume was increased to approximately 70 mL using distilled water. After 15 min of vigorous shaking and filtration, the volume was filled with distilled water until a volumetric concentration of 100 μg/mL was achieved.

#### CEF and MFX

Ten Moxinow® Tablet (400 mg CEF & 400 mg MFX/Tablet) were finely ground and weighted. A precise weight measurement was used to determine the appropriate amount of powder, equivalent to 10 mg of FPV. The powder was then transferred to a 100-mL volumetric flask, and the volume was increased to approximately 70 mL using distilled water. After 15 min of vigorous shaking and filtration, the volume was filled with distilled water until a volumetric concentration of 100 μg/mL was achieved.

### Favipiravir, cefixime and moxifloxacin hydrochloride (co-formulated)

The fixed-dose combination was formulated because FPV, CEF, and MFX fixed-dose tablets were not readily available. Ten Tablets of each pharmaceutical preparation including Favipiravir® tablets (400 mg/tablet) and Moxinow® tablets (400 & 400 mg/tablet) were weighted, finely powdered and mixed well and calculating the average weight has been done. We weighed amount of powder containing (10 mg for FPV, 10 mg for CEF and 10 mg for MFX) and transferred it to a 100-mL volumetric flask, after which the volume was diluted with distilled water to approximately 70 mL. After 15 min of vigorous shaking, the volume was completed to 100 mL with distilled water and then filtered to obtain a concentration of (100 μg for FPV, 100 μg for CEF and 100 μg for MFX per mL). Using the proposed methods, the FPV, CEF, and MFX contents were determined.

### Procedure for determination of FPV, CEF and MFX in spiked human plasma

Various aliquots (0.3, 0.4, 0.5, 0.6, 0.7 mL) of FPV, CEF and MFX standard solutions (100 μg/mL) were pipetted and transferred to 10 mL centrifuge tubes that already contained 1 mL of drug-free plasma. Then, add 3 mL of methanol to denaturate the protein. After mixing the contents of centrifuge tubes with a vortex shaker, the tubes were centrifuged for 30 min at 4000 rpm. The resulting protein-free supernatants were evaporated to dryness using a rotary evaporator under vacuum, then reconstituted in distilled water, placed in 10-mL volumetric flasks, and then the volume was diluted to 10 mL with distilled water. For each drug, the overall method was repeated with aliquots encompassing the working concentration range. Using the proposed methods, the FPV, CEF, and MFX contents were determined.

## Results and discussion

### Spectral characteristics

**Fig. 2:**
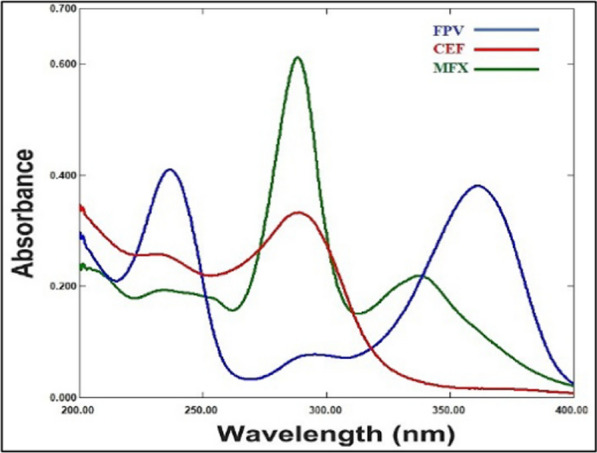
Absorption spectra of 5 μg/mL FPV, 5 μg/mL CEF and 5 μg/mL MFX

The FPV, CEF and MFX UV spectral characteristics were measured between 200 and 400 nm in wavelength. After taking a quick look at these spectra, Fig. 2 illustrates a severe overlap that explains the difficulty in directly determining that drugs simultaneously. Thus, we utilized two chemometric assisted calibration methods, namely PLS and GA-PLS, to address such overlap and determine FPV, CEF and MFX concurrently in their pharmaceutical dosage form and spiked human plasma.

### PLS and GA-PLS

The spectral matrix of the calibration data was fitted with the PLS model, a popular regression model, to infer it into new dimensions known as latent variables (LVs). PLS model was used to design a calibration model between the concentration of the studied drugs and the latent variables of the data matrix. Its ability to use all of the information in the recorded spectral data ensures greater accuracy for the spectral analysis. Additionally, PLS model has the advantage of choosing the most informative variables and excluding the uninformative ones which improves the quality of the applied model. A calibration set of 13 calibration spectra was used in conjunction with the cross validation approach, which involves removing samples one at a time, to determine the number of factors in the PLS algorithm. Consequently, the root-mean-square error cross-validation (RMSECV) was calculated after a series of LVs were gradually added to the model. Using Haaland and Thomas’s criteria [36], the best number of latent variables was chosen. The model with the best latent variable shows no statistically significant difference the corresponding root mean squares error of cross-validation and the minimum root mean squares error of cross-validation.

**Fig. 3:**
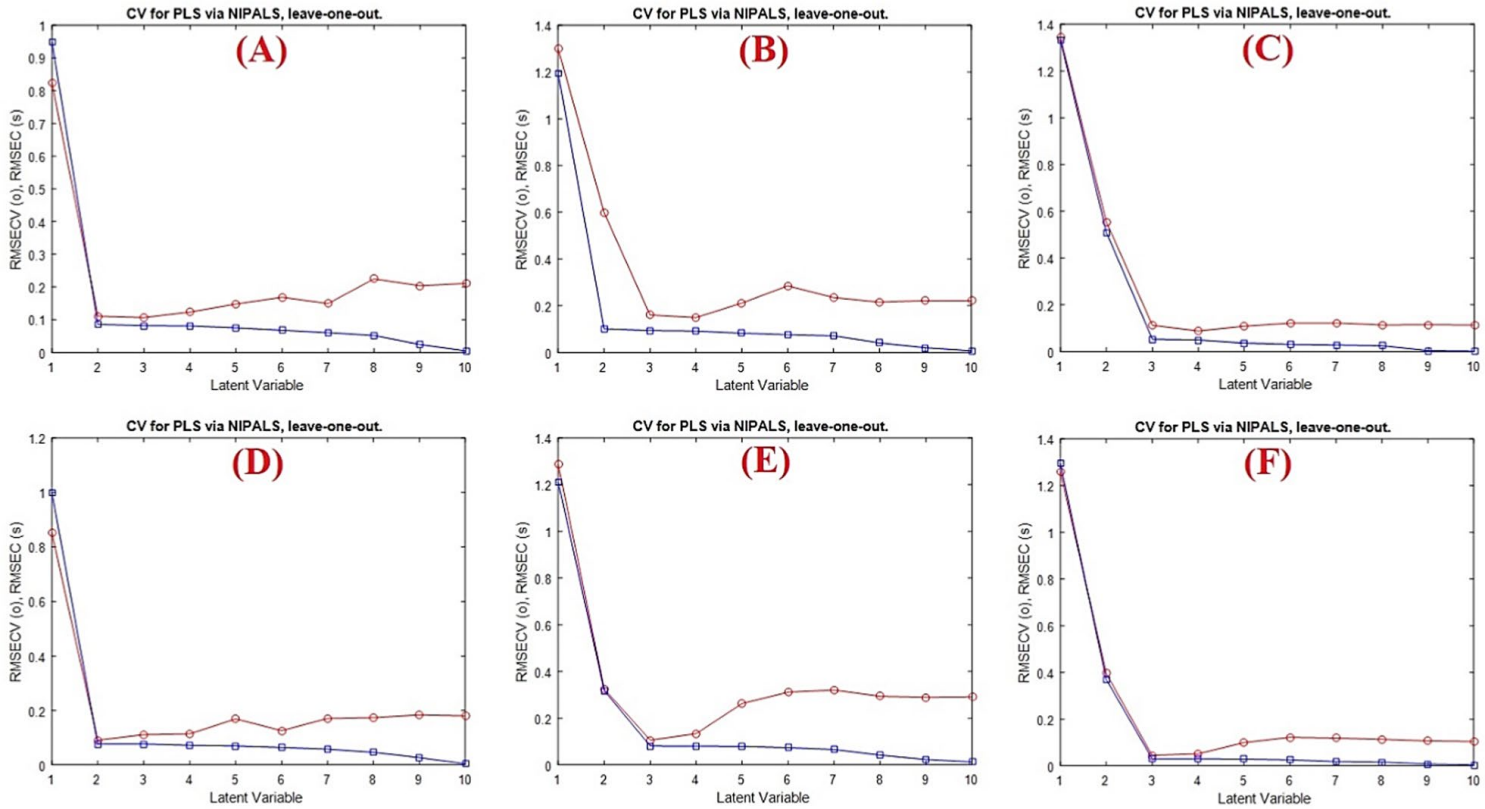
Cross validation results of the full PLS models for **A** FPV, **B** CEF, **C** MFX and the GA-PLS models for **D** FPV, **E** CEF, **F** MFX. The optimum number of latent variables shows significant decrease in their RMSECV values

As shown in Fig. 3, it was discovered that two latent variables were optimal for FPV and three latent variables for CEF and MFX with RMSECV values of 0.110, 0.160 and 0.111, respectively.

Fascinatingly, the GA procedure was employed as an informative variable’s selection technique in order to increase the PLS model’s predictive ability. To eliminate irrelevant variables while retaining informative ones, the GA model was applied to 201 variables for FPV, CEF and MFX (200–400 nm). A key factor in achieving successful GA performance is the modification of GA parameters, as indicated in Table 2. When using GAs, one of the most important factors is the population size. Choosing the right population size is an intricate issue. Larger population sizes are able to search larger spaces, which leads to an early convergence to the solution, while smaller populations perform poorly due to their limited ability to search the solution space [37]. Another crucial feature of GA was its rate of mutation, which changed one or more GA chromosomes’ genes to maintain the diversity of genetic populations and impede rapid convergence. It was discovered that the appropriate mutation rate for every medication was 0.005. Other parameters are the maximum number of LVs using the full PLS model, the number of subsets, and the number of cross-validation iterations at each generation were also estimated. Fascinatingly, it was discovered that GA reduces the absorbance matrix to roughly 33% for FVP, 25% for CEF and 23% for MFX (66 variables for FVP, 50 variables for CEF and 46 for MFX). As indicated in Tables 3 and 4, it is interesting to note that the GA-PLS models for the three drugs have lower values in terms of standard deviation (SD) of the % recoveries when compared to the full model.

**Table 2:** Optimized parameters implemented for the GA applied for variable selection selected for FPV, CEF, and MFX full spectral data

Parameters	Optimum values		
	FPV	CEF	MFX
Population size	64	64	64
Maximum generations	100	100	100
Mutation rate	0.005	0.005	0.005
% wavelength used at initiation	20	20	20
The number of variables in a window (window width)	2	2	2
Percent of population (% of convergence)	80	80	80
Cross-type	Double	Double	Double
Maximum number of latent variables	2	3	3
Cross-validation	Random	Random	Random
Number of subsets to divide data into for cross-validation	13	13	13
Number of iterations for cross-validation at each generation	2	2	2

**Table 3:** Different statistical parameters for FPV, CEF, and MFX in the calibration set by the described models

Calibration mixture	PLS			GAPLS		
	FPV	CEF	MFX	FPV	CEF	MFX
1	103.13	101.68	100.64	102.91	101.28	99.88
2	101.52	99.42	100.37	100.49	98.81	100.33
3	101.32	100.36	98.83	100.54	100.91	99.71
4	97.86	98.09	100.89	97.93	98.22	100.15
5	100.98	97.66	101.59	101.10	98.98	100.72
6	97.87	99.81	100.90	98.35	100.77	99.63
7	99.72	100.59	99.73	99.35	99.54	100.40
8	99.96	99.14	100.47	100.48	99.44	100.41
9	101.00	97.09	99.96	101.83	97.18	101.77
10	96.91	101.18	98.74	97.46	101.36	98.98
11	100.81	102.93	100.04	101.06	102.29	99.82
12	101.51	97.71	99.64	101.29	96.96	99.61
13	98.58	102.34	97.28	98.72	101.20	99.10
Mean	100.09	99.85	99.93	100.12	99.77	100.04
SD	1.813	1.8791	1.133	1.631	1.688	0.728

**Table 4:** Different statistical parameters for FPV, CEF, and MFX in the validation set by the described models

Validation mixture	PLS			GAPLS		
	FPV	CEF	MFX	FPV	CEF	MFX
1	101.48	101.99	96.26	99.63	95.70	101.05
2	98.45	95.82	101.77	99.57	96.50	100.91
3	98.50	95.13	98.55	99.69	96.27	96.70
4	100.20	100.25	102.03	100.39	100.66	100.64
5	95.73	99.62	102.73	96.43	100.84	101.87
6	96.29	98.39	101.71	96.54	99.26	100.47
7	100.17	97.71	98.13	100.60	98.24	97.99
8	96.75	103.46	97.40	96.32	102.57	98.08
9	97.10	98.69	96.13	96.85	98.02	96.79
10	101.50	104.26	98.27	101.07	102.39	100.11
11	97.35	102.20	98.51	97.33	100.90	98.26
12	102.17	104.50	99.18	101.32	102.26	101.26
Mean	98.81	100.17	99.22	98.81	99.47	99.510
SD	2.235	3.159	2.288	1.958	2.491	1.828

### Models validation

The models described was validated regarding to linearity range, accuracy, precision, limits of detection (LOD), limits of quantitation (LOQ) and selectivity parameters.

### Range of linearity

Regarding the developed PLS experimental design, which took into account the concentration range of 1–15 μg/mL for FPV, 2–15 μg/mL for CEF and 1–10 μg/mL for MFX, acceptable results were obtained over this range. While the concentration range of 1–15 μg/mL for FPV, 1–15 μg/mL for CEF and 0.5–10 μg/mL for MFX shows acceptable results for the developed GA-PLS experimental design, as indicated in Table 5.

**Table 5:** Assay validation sheet of FPV, CEF and MFX by the proposed models

Validation parameters	PLS			GAPLS		
	FPV	CEF	MFX	FPV	CEF	MFX
Wavelength (nm)	200–400	200–400	200–400	200–400	200–400	200–400
Linearity range (μg/mL)	1–15	2–15	1–10	1–15	1–15	0.5–10
Slope^a^	0.993	1.005	1.001	0.992	1.013	0.999
Intercept^a^	0.039	− 0.030	− 0.008	0.045	-0.069	0.007
Coefficient of determination (r^2^)^a^	0.9969	0.9945	0.9988	0.9975	0.9960	0.9996
LOD (μg/mL)^b^	0.291	0.383	0.177	0.261	0.323	0.104
LOQ (μg/mL)^b^	0.883	1.160	0.536	0.789	0.979	0.314
RMSEC^c^	0.089	0.097	0.054	0.080	0.084	0.032
RMSEP^d^	0.137	0.173	0.118	0.133	0.131	0.097
RRMSEP^e^	2.741	3.458	2.356	2.205	2.904	2.205
BCMSEP^f^	0.014	0.029	0.013	0.012	0.021	0.012
RMSECV^g^	0.110	0.160	0.111	0.091	0.106	0.045
Accuracy (% R)^h^	99.49	100.10	100.12	100.59	98.95	99.49
Precision (% RSD)^i^						
Repeatability	1.483	1.281	1.452	1.197	1.260	1.184
Intermediate precision	1.141	1.766	1.388	1.691	1.474	1.427

^a^Data of the straight line plotted between predicted concentrations versus actual concentrations of the calibration set

^b^The LOD and LOQ calculations are based on the net analyte signals

^c^Root-mean-square error of calibration

^d^Root-mean-square error of prediction

^e^Relative root-mean-square error of prediction

^f^Bias-corrected mean square error of prediction

^g^Root mean square error of cross-validation

^h^Average of nine determinations (three concentrations repeated three times)

^i^%RSD of nine determinations (three concentrations repeated three times)

#### Limits of detection and quantitation

LOD and LOQ were calculated, and the results were listed in Table 5. The results demonstrated the sensitivity of the proposed model for drug analysis.

### Accuracy and precision

The proposed procedure was used to determine three concentration levels in triplicate that covered the linearity ranges of the three drugs (4, 5, and 6 μg/mL for each drug). The method's precision, calculated as %RSD, was evaluated by using the proposed procedure for triplicate determination of three concentration levels that covered the linearity range of each drug (4, 5, and 6 μg/mL for each drug) within one day for repeatability and on three consecutive days for intermediate precision. Excellent %R, as displayed in Table 5, proves the proposed method’s accuracy. Additionally, small RSD values, as displayed in Table 5, prove the high method’s precision. Also, a variety of validation parameters, such as root mean square error of calibration (RMSEC), root mean square error of prediction (RMSEP), and relative root mean square error of prediction (RRMSEP), had been computed and displayed in Table 5 in order to interpret the accuracy and predictive ability of the models. Additionally, the precision or variance of the prediction was measured using the bias corrected mean square error of prediction (BCMSEP) parameter (Table 5), and the best outcomes were obtained.

### Selectivity

The standard addition technique on the already analyzed pharmaceutical samples, Table 6, was also used to assess the effect of excipients on estimation of the drugs. According to Table 7, the results obtained by application of the standard addition technique demonstrate the selectivity of the method in avoiding interference from excipients.

**Table 6:** Determination of FPV, LDP and MFX in pharmaceutical preparations and co-formulated dosage form using the described models

Favipiravir® 400 mg/tablet			Moxinow® 400 & 400 mg/tablet						Co-formulated dosage form								
FPV			CEF			MFX			FPV			CEF			MFX		
Conc (μg/mL)	PLS % R^*^	GA-PLS % R^*^	Conc (μg/mL)	PLS % R^*^	GA-PLS % R^*^	Conc (μg/mL)	PLS % R^*^	GA-PLS % R^*^	Conc (μg/mL)	PLS % R^*^	GA-PLS % R^*^	Conc (μg/mL)	PLS % R^*^	GA-PLS % R^*^	Conc (μg/mL)	PLS % R^*^	GA-PLS % R^*^
3	97.65	99.31	3	100.59	101.24	3	98.94	99.45	3	101.46	100.60	3	102.21	98.17	3	98.20	101.13
4	101.13	98.42	4	99.79	100.96	4	101.36	100.87	4	99.73	98.84	4	101.59	97.69	4	97.48	100.45
5	100.46	100.15	5	97.56	98.54	5	101.81	98.39	5	101.34	100.44	5	101.46	97.44	5	99.32	102.24
6	99.52	99.83	6	101.47	101.63	6	100.48	101.17	6	100.86	99.99	6	101.39	97.38	6	98.38	101.24
7	100.78	100.95	7	98.12	99.75	7	99.65	100.86	7	101.34	100.46	7	101.51	97.48	7	98.79	101.68
Mean	99.91	99.73	Mean	99.51	100.42	Mean	100.45	100.15	Mean	100.94	100.07	Mean	101.63	97.63	Mean	98.43	101.35
%RSD	1.398	0.947	%RSD	1.653	1.261	%RSD	1.178	1.187	%RSD	0.713	0.722	%RSD	0.327	0.333	%RSD	0.698	0.656

^*^Average of three determinations

**Table 7:** Standard addition technique data for FPV, CEF and MFX using the described models

Pharmaceutical			PLS						GA-PLS					
Drug Conc (μg/mL)	PLS found (μg/mL)	GA-PLS found (μg/mL)	FPV		CEF		MFX		FPV		CEF		MFX	
			Pure added (μg/mL)	% R^*^	Pure added (μg/mL)	% R^*^	Pure added (μg/mL)	% R^*^	Pure added (μg/mL)	% R^*^	Pure added (μg/mL)	% R^*^	Pure added (μg/mL)	% R^*^
FPV (3)	(3.04)^*^	(3.02)^*^	3	97.67	3	100.33	3	99.33	3	101.33	3	99.33	3	98.67
CEF (3)	(3.07)^*^	(2.95)^*^	3.5	101.71	3.5	98.86	3.5	102.29	3.5	99.14	3.5	98.29	3.5	101.14
MFX (3)	(2.95)^*^	(3.03)^*^	4	98.75	4	101.75	4	101.50	4	100.75	4	99.25	4	99.50
Mean				99.38		100.31		101.04		100.41		98.96		99.77
%RSD				2.109		1.442		1.513		1.130		0.588		1.263

^*^Average of three determinations

### Application of the proposed models for determination of FPV, CEF and MFX pharmaceutical dosage forms

To compare the outcomes with those of the reported methods, statistics were used [23]. As shown in Table 8, the proposed approach for analyzing the drug under investigation in its pharmaceutical dosage form did not produce any statistically significant differences when the student’s t-test and the F-test were conducted at a 95% confidence level. This suggests that the suggested method is accurate and precise.

**Table 8:** Determination of FPV, CEF and MFX in pharmaceutical tablets by described model and statistical comparison with previous reported methods

Parameters	FPV			CEF			MFX		
	PLS	GA-PLS	Reported method [10]	PLS	GA-PLS	Reported method [23]	PLS	GA-PLS	Reported method [23]
Number of measurements	5	5	5	5	5	5	5	5	5
Mean % Recovery	99.91	99.73	100.33	99.51	100.42	99.77	100.45	100.15	99.36
% RSD	1.398	0.947	0.907	1.653	1.261	1.007	1.178	1.187	0.905
Variance	1.952	0.892	0.828	2.705	1.603	1.009	1.400	1.413	0.808
Student’s *t*-test * (2.306)	0.561	1.013	–	0.311	0.899	–	1.641	1.186	–
*F*-value * (6.388)	2.358	1.077	–	2.680	1.588	–	1.733	1.749	–

^*^The values in Parenthesis are tabulated values of “*t* “and “*F*” at (P = 0.05)

### Determination of FPV, CEF and MFX in spiked human plasma

The new method was successful in monitoring FPV, CEF and MFX at therapeutic levels in spiked human plasma samples because the proposed models’ linearity and detection limits, along with the mean plasma C_max_ values for FPV (12.69–41.39 μg/mL), CEF (4.7263 ± 1.2069 μg/mL) and MFX (3.56 mg/L) [38–40], allowed for this degree of determination. As shown in Table 9, the models discussed were appropriate for determining the drugs under study in human plasma without interfering with endogenous plasma matrix components.

**Table 9:** Determination of FPV, CEF and MFX in spiked human plasma by the proposed models

PLS									GA-PLS								
FPV			CEF			MFX			FPV			CEF			MFX		
Added (μg/mL)	Found^*^ (μg/mL)	% R	Added (μg/mL)	Found^*^ (μg/mL)	% R	Added (μg/mL)	Found^*^ (μg/mL)	% R	Added (μg/mL)	Found^*^ (μg/mL)	% R	Added (μg/mL)	Found^*^ (μg/mL)	% R	Added (μg/mL)	Found^*^ (μg/mL)	% R
3	2.83	94.46	3	2.91	96.84	3	2.79	92.85	3	2.76	92.12	3	2.79	92.91	3	2.90	96.66
4	3.78	94.39	4	3.73	93.34	4	3.89	97.16	4	3.80	95.01	4	3.82	95.49	4	3.79	94.65
5	4.84	96.80	5	4.75	95.09	5	4.84	96.74	5	4.84	96.75	5	4.72	94.46	5	4.83	96.55
6	5.64	93.93	6	5.89	98.10	6	5.62	93.74	6	5.63	93.90	6	5.83	97.18	6	5.65	94.20
7	6.78	96.80	7	6.80	97.08	7	6.78	96.91	7	6.78	96.85	7	6.78	96.86	7	6.80	97.08
Mean		95.28	Mean		96.09	Mean		95.48	Mean		94.93	Mean		95.38	Mean		95.83
%RSD		1.476	%RSD		1.956	%RSD		2.121	%RSD		2.104	%RSD		1.845	%RSD		1.336

^*^Average of five determinations

### Green assessment of the described models

Two new approaches to assessing the greenness of the suggested method were presented: the analytical eco-scale [34, 41] and the green analytical procedure index. The eco-scale relies on penalization points calculated from reagents, instruments, and waste to facilitate their development as semi-quantitative methods. The method relies on subtracting the total number of penalty points from 100. The higher the value of the result, the more environmentally friendly the newly developed approach [35]. In Table 10, the sum of the penalty points for the suggested technique were 3 and 9 points for application of Pharmaceutical dosage forms and Spiked human plasma, respectively that resulted a total scoring of 91 and 97. This shows that the suggested approach is just as environmentally friendly as the reported spectrophotometric method for CEF and MFX [23], but it is more environmentally friendly than the reported techniques for favipiravir [10, 42]. Using five pictograms and a unique symbol, the GAPI metrics rate how environmentally friendly each stage of the analytical process is. Every pictogram is made up of different fields and denotes a specific stage in an analytical procedure. The environmental effects of each field are classified as low, medium, and high (green, yellow, and red), and their corresponding quantities are computed. Furthermore, a specific circle indicates whether or not the approach being studied includes quantification [35].

**Table 10:** Greenness assessment of the proposed and HPLC-reported techniques utilizing the Eco-scale and GAPI tools

Parameters	Proposed Chemometric method		Reported method for FPV (HPLC) [10]	Reported method for FPV (spectrophotometry) [42]		Reported method for CEF and MFX [23]
Application	Pharmaceutical dosage forms	Spiked human plasma	Pharmaceutical dosage forms	Pharmaceutical dosage forms	Spiked human plasma	Pharmaceutical dosage forms
Reagents						
Water	0	0	–	–	–	0
Methanol	–	6	12	–	–	–
Acetonitrile	–	–	8	–	–	–
Phosphate buffer	–	–	0	–	–	–
Ethanol	–	–	–	6	6	–
Instruments						
Spectrophotometer/ /HPLC						
Energy	0 [≤ 0.1 kWh/sample]	0 [≤ 0.1 kWh/sample]	1 [> 0.1 kWh/sample]	0 [≤ 0.1 kWh/sample]	0 [≤ 0.1 kWh/sample]	0 [≤ 0.1 kWh/sample]
Occupational hazard	0	0	0	0	0	0
Waste	3	3	6	3	3	3
Total penalty points	Σ 3	Σ 9	Σ 27	Σ 9	Σ 9	Σ 3
Analytical eco-scale total score	97	91	73	91	91	97
Analytical eco-scale total score^a,b^	Excellent green analysis	Excellent green analysis	Acceptable green analysis	Excellent green analysis	Excellent green analysis	Excellent green analysis
GAPI pentagram						

^a^Analytical eco-scale total score = 100−total penalty points

^b^If the score is greater than 75, indicates that the green analysis is excellent

If the score is greater than 50, it indicates that the green analysis is acceptable

If the score is of 50 or less, it indicates insufficient green analysis

The described models for the proposed method had nine and seven green zones for application in pharmaceutical dosage forms and spiked human plasma, respectively. This indicates the proposed model has the same greenness as the previously reported spectrophotometric method for CEF and MFX [23]. Comparing with other reported methods of favipiravir, the proposed method has more green zones with the same number of red zones when applied in the same matrix. In conclusion, the green metrics' findings provided a thorough environmental friendliness profile and, for the most part, verified compliance with green practices.

## Conclusion

In the proposed study, two novel multivariate chemometric methods were used to validate a new analytical tool for the simultaneous determination of FPV, CEF and MFX in pharmaceutical preparations and spiked human plasma. Without requiring a separation step, the chemometric techniques under study demonstrated excellent sensitivity and resolving power. This in turn provides more economical alternatives, higher levels of simplicity, and faster analysis times all of which are necessary for the numerous regular daily analyses that pharmaceutical research and quality control laboratories perform. To enable integrated green spectrophotometric determination of the drugs under study, chemometric models were built and refined. The Green Analytical Procedure Index, and the analytical eco-scale were used to assess the greenness of the models. In terms of the official green metric values, the results demonstrated that the models described complied and met the environmental friendliness requirements.

## Data Availability

The datasets used and/or analyzed during the current study are available from the corresponding author on reasonable request.

## Abbreviations

FPV: Favipiravir
CEF: Cefixime trihydrate
MFX: Moxifloxacin hydrochloride
PLS: Partial least squares
GA: Genetic Algorithm
ICH: International Conference on Harmonization
GAPI: Green Analytical Procedure Index
